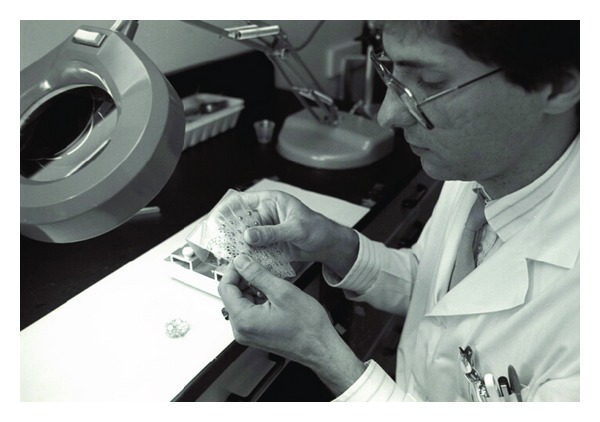# Slowly Evolving Trends in Temporal Lobe Epilepsy Management at London Health Sciences Centre

**DOI:** 10.1155/2013/387510

**Published:** 2013-02-25

**Authors:** Warren T. Blume

**Affiliations:** London Health Sciences Centre, Western University, London, ON, Canada N6A 5A5

## Abstract

Although the advent of MRI impacted significantly our presurgical investigation, ictal semiology with interictal and ictal EEG has clearly retained its roles in localizing epileptogenesis. MRI-identified lesions considered epileptogenic on semiological and electroencephalographic grounds have increased the likelihood of resective surgery effectiveness whereas a nonlesional MRI would diminish this probability. Ictal propagation and the interplay between its source and destination have emerged as a significant component of seizure evaluation over the past 30 years.

## 1. Seizure Semiology and Epilepsy Evaluation before and Since MRI

Ictal semiology and EEG dominated our localization of intractable epileptogenesis prior to the introduction of MRI. Dr. John Girvin and I each attempted to outdo the other in obtaining patient and observer descriptions of the patients' seizures, guided by the most comprehensive observations and perceptions documented by Wilder Penfield and Herbert Jasper in *Epilepsy and Functional Anatomy of the Human Brain *[1]. Seizures of the first 3 medically intractable patients operated upon at University Hospital, London originated in the frontal, occipital, and anterior parietal lobes. Similar extratemporal experiences in Montreal and Glasgow (Dr. Girvin) and the Mayo Clinic (WTB) paradoxically sharpened our clinical definition of temporal/limbic epilepsy. 

In addition to Penfield's identification for a semiological pattern as representing temporal lobe epilepsy and subsequent description of mesial temporal ictal semiology [2], subsequent studies disclosed that some features such as version and dysphasia could lateralise epileptogenesis within the temporal lobe, enhancing further the semiological role in this evaluation [3, 4].

The works of International League against Epilepsy Commissions on Epileptic Seizure Classification and Terminology have, in sequential fashion, clarified our clinical analyses. The 1981 ILAE Commission classified partial (focal) seizures into simple partial (consciousness preserved) and complex partial (consciousness impaired). This division has encountered clinical and heuristic limitations as it depends upon evaluating an entity—consciousness—that can neither be defined nor assessed. 

Gloor [5] discusses the several aspects of consciousness presented by philosophers (1713), neuropsychologists, and other neuroscientists and since Hebb [6]. “As none of the attempts at arriving at a scientifically satisfactory concept of consciousness have been successful” [5], neuroscientists have turned to the more tractable aspects of “consciousness” such as perception, memory, affect, and voluntary movements [6]. Continuing in this direction, the ILAE replaced consciousness and “complex partial” with “cognition” as the pivotal defining concept of focal seizures by creating as “dyscognitive” a focal or generalised seizure type that impairs two or more aspects of cognitive function. Thus “dyscognitive” refers to a seizure in which a disturbance of cognition is the predominant or most apparent feature. Components of cognition are all assessable and include: perception, attention, emotion, memory, and executive function [7]. Guided by neuropsychologists, clinicians, health care staff, relatives, or other observers could intraictally administer specific tests for each of these possible components and thus, over several attacks, fully characterize a dyscognitive seizure disorder. For example, unresponsiveness could represent ictal dysphasia or dyspraxia. No recall of an ictus with intact responses may be a “pure amnestic seizure” [8].

## 2. Electroencephalography (EEG)

Combined with ictal semiology, ictal and interictal EEG were the principal tools before the mid-1980s to localize epileptogenesis in virtually all patients whose intractable focal epilepsies required resective surgery for alleviation. Focal temporal interictal spikes, if predominant over one temporal lobe, lateralised temporal lobe seizure origin in over 90% of patients [9]. As the ictal scalp EEG is often marred by scalp muscle, movement, and electrode artifact, such high correlation of interictal EEG epileptiform abnormalities with temporal seizure origin degraded the long-held ictal EEG as the “gold standard” of identifying seizure origin.

For many years, nasopharyngeal or sphenoidal EEG leads supplemented the Ten Twenty EEG electrode system to better record anterior-mesial temporal EEG activity, especially spikes. As illustrated by F. A. Gibbs and E. L. Gibbs [10], the anterior temporal spike field was usually centred below the Ten Twenty Electrodes [11]. When Sadler and Goodwin [12] demonstrated convincingly that mandibular notch electrodes recorded anterior temporal spikes just as well as sphenoidal leads, we abandoned the latter for reasons of ease of application and patient comfort.

Our group at Western/University Hospital was the first to formally study and describe the morphology of scalp-recorded focal seizures-temporal and extratemporal [13]. Recognition of such features sharpened our visual assessment of clinical seizures.

## 3. Subdural EEG

In 1979, Dr. John Girvin and Mr. Dan Jones, EEG technologist, designed *subdural electrodes. *Inserted as imbedded in silicon strips through burr holes to avoid a craniotomy during the patient's evaluation, these electrodes record directly from the cortical surface. Moreover, they may extend to mesial and inferior cortical surfaces, areas remote from scalp electrodes. For patients with temporal lobe epilepsy, SDE provides more precise and sensitive ictal and interictal recording from the mesial temporal regions. 

**Figure 1:**
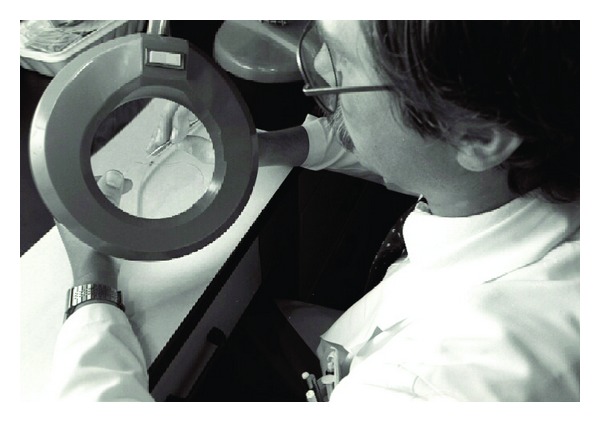


Commercially manufactured subdural electrodes were not available in the early 1980s, resulting in some other epilepsy centres purchasing our in-house-manufactured electrodes. Design and manufacture of SDE at UH without any complication continued by Dan Jones and Frank Bihari for over 20 years. Figures 1 and 2 depict Mr. Jones and Mr. Bihari designing and inspecting our subdural electrodes.

However, in 2004 London Health Sciences Centre chose to make use of commercially produced electrodes, though at a significantly greater cost. 

Our centre was the first to routinely employ subdural electrodes (SDE), and they continue today as a major part of evaluation of about 50% of our patients ultimately operated upon.

 We studied 27 consecutive patients whose temporal lobe epilepsy clinically implicated both temporal lobes from ictal semiology, scalp EEG, and imaging features. We found that the side of SDE-recorded seizures correlated with that containing most scalp spikes and most scalp-recorded seizures in most but not all patients, confirming the value of both EEG and SDE [14].

## 4. Pre-MRI Imaging

Although unable to detect small cortical epileptogenic lesions, plain skull X-rays had disclosed several cranial and intracranial abnormalities of lateralising value. Cranial erosion on skull roentgenography could have resulted from a previous wide fracture or from a subdural or subarachnoid cyst; one or more asymmetrical cranial features may have indicated cerebral hemiatrophy, compatible with accompanying epileptogenesis; intracerebral calcification may have represented tumours or congenital lesions. Pneumoencephalography disclosed displaced or deformed ventricles from tumours, abscesses, hematoma, and other lesions. Cerebral arteriography helped localize expanding lesions but proved less helpful in the study of atrophic lesions [1]. 

## 5. Magnetic Resonance Imaging (MRI)

A major advance in evaluating patients for temporal lobe surgery was the advent of MRI. Imaging afforded by MRI discloses focal structural abnormalities underlying intractable epilepsy that may remain undetected by earlier neuroimaging methods [15]. Subsequently, Lee et al. [16] demonstrated both high sensitivity and specificity for MRI in detecting pathologically verified hippocampal/amygdala and other temporal lobe epileptogenic lesions. 

Three common epileptogenic lesions are particularly well displayed by MRI: mesial temporal sclerosis, cortical dysplasia (CD), and benign tumours such as dysembryoplastic neuroepithelial tumours (DNETs) and gangliogliomas (GGL). Rhythmic epileptiform discharges (REDs) may characterize the scalp EEGs of those with focal CD [17]; abundant focal spikes appear on scalp EEGs of DNET and GGL patients.

The presence or absence of mesial temporal sclerosis (MTS) became far better displayed by MRI than any previous imaging modality. This greatly facilitated, with EEG, an assessment as to whether one or both temporal lobes are epileptogenic. In the latter instance, assessment of their relative epileptogenicities may influence a surgical decision. 

In some patients, MRI and EEG (via REDs) have demonstrated both MTS and CD with comparable epileptogenicity in each, termed “dual pathology” [18, 19]. Originally we opted for temporal lobectomy as the mesial temporal epilepsy is the region most likely to resist AED therapy [20]. However subsequent experience has suggested that a cortical CD or similar lesion should be resected first.

The appearance of dual pathology in some patients augmented our awareness of seizure propagation and its influence on semiology. Thus, ictal symptoms and signs may signify the site of seizure spread rather than origin. Both normal cerebral connectivity and neuronal pathways developed in the process of epileptogenesis likely participate in seizure spread. For example, occipital seizures may propagate to the mesial temporal region via the “ventral stream,” a multisynaptic pathway that terminates primarily in the amygdala but also in the parahippocampal area [21]. Munari and Bancaud [22] described ictal fear, epigastric and olfactory sensations, and oroalimentary automatisms consequent to seizure spread from the orbital frontal cortex to the amygdala and insula. Suspect this situation in any patient with: (1) intractable limbic-like seizures and no anterior-mesial temporal EEG spikes, (2) no temporal MRI pathology, and (3) frontal lobe semiology in some seizures.

## 6. Neurosurgical Approach and Technique

Three approaches to the mesial temporal region have been described: sylvian fissure, middle temporal gyrus, and inferior temporal pole [23]. Our surgeons have accessed the mesial temporal structures via the middle temporal gyrus (Steven DA, *personal communication*). However, often a full temporal lobectomy has been performed (Parrent A, *personal communication*). Surgical approach and technique have not measurably changed over the years.

## 7. Outcome: Neuropsychological Effects

That a substantial left temporal lobectomy will significantly impair verbal memory has become increasingly realized over the past decades creating a distinction between left and right temporal epilepsy in terms of surgical candidature [24, 25]. Concern about verbal memory and other verbal functions has raised considerably our threshold for left temporal lobectomy over the past several years.

## Figures and Tables

**Figure 2:**